# The effect of optimism, pessimism, and jealousy on emotional regulation in athletes

**DOI:** 10.3389/fpsyg.2026.1862982

**Published:** 2026-09-24

**Authors:** Mehmet Aydoğan, Yunus Şahinler, Taner Atasoy

**Affiliations:** Faculty of Sports Sciences, Istanbul Gelisim University, Istanbul, Türkiye

**Keywords:** athletes, emotion regulation, jealousy, mediation analysis, optimism, pessimism

## Abstract

**Introduction:**

The aim of this study was to examine the distinct associations of optimism and pessimism with emotion regulation in athletes and to test whether jealousy mediates each association.

**Methods:**

The sample comprised 628 athletes from the Ataşehir and Bakırköy districts of Istanbul (mean age = 25.73). Data were collected using the Athlete Emotion Regulation Scale (AERS), the Athlete Optimism and Pessimism Scale (AOPS), and the Jealousy Scale for Athletes (JSA). Because AOPS is a two-factor instrument, optimism and pessimism were treated as separate subscale scores rather than combined into a single predictor. Two parallel mediation analyses were specified with Hayes PROCESS Model 4, with optimism or pessimism as the predictor (X), jealousy as the mediator (M), and emotion regulation as the outcome (Y).

**Results:**

In the optimism model, optimism negatively predicted jealousy (B = −0.52, *p* < 0.001) and positively predicted emotion regulation after controlling for jealousy (B = 2.20, *p* < 0.001); the indirect effect through jealousy was negative and significant (B = −0.70, bootstrap 95% CI [−0.8232, −0.5982]). In the pessimism model, pessimism positively predicted jealousy (B = 1.50, *p* < 0.001) and showed a negative direct association with emotion regulation (B = −2.06, *p* < 0.001); the indirect effect through jealousy was approximately B = 5.27 and was reported as significant based on 5,000 bootstrap samples (the 95% bootstrap confidence interval excluded zero).

**Discussion:**

These findings indicate that optimism and pessimism follow distinct pathways and should not be interpreted as a single combined psychological construct.

## 1 Introduction

Optimism, pessimism, jealousy, and emotion regulation are conceptually distinct psychosocial constructs that may shape athletes’ adaptation to competitive demands (Galli and Gonzalez, 2015; Lane et al., 2011). Optimism generally reflects favorable expectations about future outcomes, whereas pessimism reflects unfavorable expectations; the two constructs may be related but should not be assumed to represent the same score or to increase simultaneously. The present study also uses the term jealousy consistently for the construct measured by the Jealousy Scale for Athletes (JSA). Jealousy and envy are related social-comparison emotions but are conceptually distinct (Smith and Kim, 2007; van de Ven et al., 2009; Lange and Crusius, 2015; Vrabel et al., 2021), and are therefore not used interchangeably in this manuscript (Parrott, 1991; Harris and Darby, 2010).

In sport settings, optimism has been associated with favorable psychological and performance-related outcomes (Hussain et al., 2023). Dandekar et al. (2017) examined college students participating in individual sports and linked dispositional optimism with sport-related psychological characteristics. In a sample of 227 national-level wrestlers, Berengüí et al. (2013) reported that more optimistic athletes showed lower burnout-related symptoms. Beyond sport, Lee et al. (2019) and Zou et al. (2022) demonstrated that optimism and pessimism can show differential associations with reappraisal and acceptance, reinforcing the need to examine the two orientations separately rather than as a single undifferentiated score.

Pessimism is generally associated with less adaptive responses to stress and may be particularly relevant in sport environments characterized by repeated evaluation, training load, and competitive pressure. Burnout research in competitive sport has linked less favorable dispositional orientations with emotional exhaustion and reduced accomplishment (Berengüí et al., 2013), while longitudinal observations in volleyball players emphasize that coping and burnout perceptions vary across the competitive season (Pires and Ugrinowitsch, 2021). These findings support examining pessimism as a distinct predictor with potentially different associations from optimism.

Jealousy in sport can emerge in contexts involving competition, social comparison, status, resources, and interpersonal relationships. Evidence from elite youth sport and interuniversity student-athletes shows that sibling and family dynamics can shape athletes’ psychological experiences across development (Ferguson and Strachan, 2017; Lundy et al., 2019; Hopwood et al., 2015). The JSA was specifically developed for licensed athletes and operationalizes jealousy across performance and achievement, conditions and opportunities, and social support and popularity domains (Çekiç et al., 2023). These domains make jealousy particularly relevant to social-comparison processes in competitive sport.

The theoretical model was therefore reformulated to preserve the distinction between optimism and pessimism (Scheier and Carver, 1985). Social comparison theory proposes that evaluations of the self-relative to others can generate affective responses in achievement contexts (Festinger, 1954 Tesser et al., 1988; Cikara and Fiske, 2011), while emotion-regulation research indicates that future-oriented expectations are related to the strategies individuals use to manage emotional experiences (Carver et al., 2010; Zou et al., 2022). On this basis, jealousy was conceptualized as a potential mediating process linking each AOPS subscale separately with emotion regulation. This specification avoids treating high optimism and high pessimism as a single simultaneous state.

The purpose of this study was to examine optimism and pessimism as separate predictors of athletes’ emotion regulation and to test the mediating role of jealousy in each relationship. Accordingly, two parallel mediation models were evaluated: Optimism → Jealousy → Emotion Regulation and Pessimism → Jealousy → Emotion Regulation.

In this context,

H1a: Optimism is negatively associated with jealousy in athletes; H1b: pessimism is positively associated with jealousy in athletes.

H2a: Optimism is positively associated with emotion regulation scores; H2b: pessimism is negatively associated with emotion regulation scores.

H3: Jealousy is significantly associated with emotion regulation scores in athletes.

H4a: Jealousy mediates the association between optimism and emotion regulation; H4b: jealousy mediates the association between pessimism and emotion regulation.

## 2 Materials and methods

### 2.1 Research model

This study used a cross-sectional correlational design to examine associations among optimism, pessimism, jealousy, and emotion regulation (Karasar, 2007, 2012). The mediation analyses were used to evaluate indirect statistical associations and were not interpreted as establishing temporal or causal effects. Optimism and pessimism were modeled as separate predictor variables in accordance with the two-factor structure of the AOPS.

### 2.2 Participants

The study population comprised individuals actively participating in sport in Istanbul, and the sample included 628 athletes from multiple sport branches in the Ataşehir and Bakırköy districts. The mean age was 25.73 years; 54.3% were male (*n* = 341) and 45.7% were female (*n* = 287). By age group, 40.0% were 18–23 years (*n* = 251), 35.0% were 24–29 years (*n* = 220), and 25.0% were 30 years or older (*n* = 157). Sport branch was included in the personal information form; however, branch-specific frequency counts were not retained in the analysis output and therefore could not be reported. Competition level was not included in the personal information form and therefore could not be used to characterize the sample.

### 2.3 Data collection procedure

Data were collected cross-sectionally from the 628 athletes in the Ataşehir and Bakırköy districts using a self-report questionnaire package comprising the personal information form, the Athlete Emotion Regulation Scale, the Athlete Optimism and Pessimism Scale, and the Jealousy Scale for Athletes. Detailed information regarding the recruitment channel, exact administration setting, data-collection dates, and whether the questionnaires were completed individually or in groups was not retained in the available study records; consequently, these characteristics could not be reported.

### 2.4 Data collection tools

In this section, the personal information form created by the researcher, the Athlete Emotion Regulation Scale, the Athletes’ Optimism and Pessimism Scale, and the Jealousy Scale for Athletes were used.

#### 2.4.1 Personal information form

The personal information form created by the researchers included gender, age, and sport branch. Competition level was not included in this form.

#### 2.4.2 Athlete emotion regulation scale

The Emotion Regulation Scale was developed by Gross and John (2003), adapted into Turkish by Eldeleklioğl and Eroğlu (2015), and adapted specifically for athletes by Tingaz and Altun-Ekiz (2021). The athlete form contains eight items rated on a seven-point Likert scale and comprises two factors: Suppression (items 1, 2, 4, and 7) and Cognitive Reappraisal (items 3, 5, 6, and 8). In the athlete adaptation study, the internal structure was examined through both exploratory and confirmatory factor analyses in athlete samples, and the original two-factor structure was retained. The reported Cronbach’s alpha coefficients were 0.65 for Suppression and 0.73 for Cognitive Reappraisal (Tingaz and Altun-Ekiz, 2021).

#### 2.4.3 Athlete optimism and pessimism scale

Developed by Tekkurşun-Demir (2022), the Athlete Optimism and Pessimism Scale (AOPS) contains 18 items rated on a five-point Likert scale and consists of two distinct subscales. Items 1–9 form the optimism subscale (score range 9–45; α = 0.81), whereas items 10–18 form the Pessimism subscale (score range 9–45; α = 0.92). In the present analyses, these subscales were scored and modeled separately; no combined optimism–pessimism total score was interpreted.

The original AOPS validation study used exploratory factor analysis (EFA), confirmatory factor analysis (CFA), and convergent/divergent validity procedures in athlete samples. The two-factor solution explained 57.77% of the total variance, with the optimism factor explaining 41.15% and the Pessimism factor 15.62%, and the two-factor structure was subsequently examined by CFA (Tekkurşun-Demir, 2022).

#### 2.4.4 Jealousy scale for athletes (JSA)

Developed by Çekiç et al. (2023), the Jealousy Scale for Athletes (JSA) assesses jealousy among athletes using three dimensions: Performance and Achievement Jealousy, Conditions and Opportunities Jealousy, and Social Support and Popularity Jealousy. The scale contains 21 items rated on a five-point Likert scale. During scale development, exploratory factor analysis was conducted in an athlete sample and the three-factor structure was subsequently tested with confirmatory factor analysis in a separate licensed-athlete sample. The reported Cronbach’s alpha coefficients for the three dimensions were 0.858, 0.859, and 0.868, respectively (Çekiç et al., 2023).

### 2.5 Ethical approval

Ethical approval for this study was obtained from the Ethics Committee of Istanbul Gelişim University (Decision No: 2025-1185).

### 2.6 Data analysis

The data were analyzed using SPSS 26.0 and Hayes’ PROCESS macro (Hayes, 2022). Descriptive statistics, distributional checks, and reliability analyses were examined before hypothesis testing. Because AOPS contains two conceptually distinct subscales, optimism and pessimism were not combined. Two separate mediation analyses were estimated with PROCESS Model 4 using 5,000 bootstrap samples: Model 1 specified Optimism as X, Jealousy (JSA) as M, and Emotion Regulation (AERS) as Y; Model 2 specified Pessimism as X, JSA as M, and AERS as Y. Unstandardized regression coefficients (B), standard errors, p values, and 95% confidence intervals were used for reporting the regression paths, and an indirect effect was considered significant when its bootstrap 95% confidence interval did not include zero. This specification makes the analytical sequence explicit and avoids redundant simple and multiple regression tests that duplicate paths already estimated within PROCESS Model 4. Additional descriptive figures and detailed regression and mediation outputs are provided in the Supplementary material.

## 3 Results

Among the 628 athletes, 341 (54.3%) were male and 287 (45.7%) were female.

Regarding age, 251 participants (40.0%) were 18–23 years, 220 (35.0%) were 24–29 years, and 157 (25.0%) were 30 years or older.

The revised analyses treat optimism and pessimism as separate AOPS subscales. For JSA and AERS, the skewness/kurtosis values were 0.856/1.057 and −0.639/1.095, respectively, both within the range treated as acceptable in the original analysis. The previously reported AOPS value (−0.327/0.952) referred to a combined score and was therefore not interpreted as a distributional statistic for either the Optimism or Pessimism subscale. Indirect-effect inference was based on 5,000 bootstrap samples in PROCESS Model 4.

### 3.1 Mediation model 1: Optimism → Jealousy → Emotion Regulation

In the optimism model, Optimism significantly and negatively predicted Jealousy, B = −0.52, SE = 0.05, *t* = −10.24, *p* < 0.001, 95% CI [−0.62, −0.42], with R^2^ = 0.16 for the mediator model. In the outcome model, Optimism significantly and positively predicted AERS scores after Jealousy was controlled, B = 2.20, SE = 0.08, *t* = 27.14, *p* < 0.001, 95% CI [2.04, 2.36], and Jealousy also positively predicted AERS scores, B = 1.35, SE = 0.06, *t* = 21.68, *p* < 0.001, 95% CI [1.23, 1.47]. The outcome model explained 61.7% of the variance (R^2^ = 0.62). The total effect of Optimism on AERS was positive (B = 1.49, *p* < 0.001), whereas the indirect effect through Jealousy was negative and significant (B = −0.70, bootstrap 95% CI [−0.8232, −0.5982]), indicating an inconsistent/suppressing mediation pattern.

### 3.2 Mediation model 2: Pessimism → Jealousy → Emotion Regulation

In the pessimism model, Pessimism significantly and positively predicted Jealousy (B = 1.50, *p* < 0.001). In the outcome model, Pessimism showed a negative direct association with AERS scores, B = −2.06, SE = 0.49, *t* = −4.21, *p* < 0.001, 95% CI [−3.02, −1.10], whereas Jealousy positively predicted AERS scores, B = 3.51, SE = 0.31, *t* = 11.04, *p* < 0.001, 95% CI [2.88, 4.13]. The total effect of Pessimism on AERS was B = 3.21 (*p* < 0.001). Based on the rounded coefficients reported in the analysis, the indirect effect through Jealousy was approximately B = 5.27 and was significant in the 5,000-sample bootstrap analysis, with the 95% bootstrap confidence interval excluding zero. The opposite signs of the direct and indirect effects indicate an inconsistent mediation pattern.

## 4 Discussion

The revised analysis treats optimism and pessimism as distinct predictors and evaluates two parallel mediation models with jealousy as the mediator and emotion regulation as the outcome. This distinction is consistent with the validated two-factor structure of AOPS and prevents the conceptual problem of interpreting simultaneously high optimism and high pessimism as one combined state (Tekkurşun-Demir, 2022).

For optimism, higher scores were associated with lower jealousy and with higher AERS scores after jealousy was controlled. The negative association between optimism and jealousy is compatible with the proposition that favorable future expectations may reduce threatening social-comparison appraisals. However, because jealousy was positively associated with AERS scores in the outcome model, the indirect pathway was negative and partially offset the positive direct association of optimism. This pattern is more appropriately described as inconsistent or suppressing mediation rather than as a uniformly positive or negative mediated effect.

For pessimism, higher scores were associated with greater jealousy, while the direct association between pessimism and AERS scores was negative. This direction is consistent with the broader literature linking pessimistic expectations with less adaptive responses to stress (Carver et al., 2010; Zou et al., 2022). At the same time, the positive Jealousy → AERS coefficient produced an indirect pathway with the opposite sign to the direct pessimism effect. Accordingly, the findings should be interpreted as evidence of competing statistical pathways rather than as evidence that increases in optimism and pessimism jointly produce the same emotional-regulation outcome.

The manuscript consistently refers to jealousy, not envy, because jealousy is the construct operationalized by the JSA. Social comparison theory provides a useful framework for understanding why jealousy may emerge in competitive settings where athletes compare performance, recognition, resources, and social support (Festinger, 1954; Salovey and Rodin, 1984). Nevertheless, the positive association between JSA and AERS scores should not be interpreted automatically as evidence that jealousy improves emotion-regulation ability; it indicates only the direction of association with the measured AERS score in the present cross-sectional model.

Taken together, the two mediation models show why optimism and pessimism must be analyzed separately. The optimism model contained a negative indirect pathway through jealousy, whereas the pessimism model contained a significant indirect pathway that opposed the negative direct effect. These distinct coefficient patterns would be obscured by a single combined optimism–pessimism predictor. Because the design is cross-sectional, the mediation findings describe statistical indirect associations and do not establish causal or temporal mechanisms.

### 4.1 Limitations

This study has several limitations. First, the cross-sectional design limits causal and temporal inference regarding optimism, pessimism, jealousy, and emotion regulation. Second, all variables were assessed using self-report measures and may therefore be influenced by response and social-desirability biases. Third, the sample was restricted to athletes from Ataşehir and Bakırköy in Istanbul, limiting generalizability across regions and sport systems. Competition level was not included in the personal information form, which limits interpretation across recreational, amateur, professional, or elite categories. In addition, the AERS contains distinct emotion-regulation strategies; therefore, associations with the overall AERS score should be interpreted as score-level relationships rather than as unequivocal improvements or impairments in emotion-regulation ability. Future studies should use longitudinal designs, report detailed sport-branch and competition-level characteristics, and examine the model across different sport contexts.

## 5 Conclusion

In conclusion, optimism and pessimism showed distinct associations with jealousy and emotion regulation and should not be combined into a single predictor. In the optimism model, higher optimism was associated with lower jealousy and a positive direct association with AERS scores, while the indirect pathway through jealousy was negative. In the pessimism model, higher pessimism was associated with greater jealousy and a negative direct association with AERS scores, while the significant indirect pathway operated in the opposite direction. These findings highlight the importance of preserving the theoretical and psychometric distinction between optimism and pessimism when examining emotional processes in athletes.

The findings also emphasize that jealousy should be interpreted as a distinct social-comparison emotion rather than used interchangeably with envy. From an applied perspective, psychological skills programs may benefit from considering athletes’ future-oriented expectations and social-comparison experiences separately rather than treating positive and negative expectations as a single continuum.

Any practical interpretation should remain cautious because the observed pathways are cross-sectional and because higher AERS scores do not necessarily indicate uniformly more adaptive regulation across all strategies. Interventions should therefore be informed by the specific regulation strategy, the athlete’s optimistic or pessimistic orientation, and the social context in which jealousy occurs.

Future research should replicate the two separate mediation models in diverse sport branches and competition levels, use longitudinal or experimental designs where feasible, and examine the cognitive reappraisal and suppression dimensions of emotion regulation separately. Qualitative approaches may also clarify how athletes experience jealousy and social comparison in training and competition.

## Data Availability

The datasets generated and/or analyzed during the current study are not publicly available because the participants did not provide consent for their data to be deposited in a public repository. However, the anonymized data supporting the findings of this study are available from the corresponding author upon reasonable request. Requests to access the datasets should be directed to YŞ (ysahinler@gelisim.edu.tr).
