# Supplementary material for: The effect of optimism, pessimism, and jealousy on emotional regulation in athletes
Source: Front Psychol. 2026 Sep 24;17:1862982. doi: 10.3389/fpsyg.2026.1862982 (PMC13645547; doi:10.3389/fpsyg.2026.1862982)
Supplement: Supplementary file 1 [file Supplementary_file_1.docx]

Supplementary Material

Revision color code: Reviewer 1 revisions = red; Reviewer 2 revisions = blue.

**Figure 1. Distribution of Participants by Gender (n = 628)**

**Figure 2. Distribution of Participants by Age Group (n = 628)**

**Table 1.** Skewness and Kurtosis Values of Variables
*The combined AOPS value is retained only as the original descriptive distributional check; it was not used as a predictor in the revised mediation analyses. Optimism and Pessimism were modeled separately.

| **Variable** | **Skewness** | **Kurtosis** |
| --- | --- | --- |
| AOPS combined score* | *-0,327* | *0,952* |
| JSA | 0,856 | 1,057 |
| **AERS** | *-0,639* | *1,095* |

**Figure 3. PROCESS Model 4 for Optimism** as the predictor, Jealousy (JSA) as the mediator, and Emotion Regulation (AERS) as the outcome.

**Figure 4. PROCESS Model 4 for Pessimism** as the predictor, Jealousy (JSA) as the mediator, and Emotion Regulation (AERS) as the outcome.

**Table 2.** Regression Analysis Results Regarding the Effect of Optimism Scale Score on Jealousy Scale Score

| **Variable** | **B** | **SE** | **t** | **p** | **95% CI Lower** | **95% CI Upper** | **R** | **R²** | **F** | **p (model)** |
| --- | --- | --- | --- | --- | --- | --- | --- | --- | --- | --- |
| **Constant** | 4.2792 | 0.1751 | 24.4370 | <.001 | 3.9352 | 4.6231 | .4018 | .1614 | 104.92 | <.001 |
| Optimism | –0.5211 | 0.0509 | –10.2431 | <.001 | –0.6210 | –0.4212 |  |  |  |  |

***Note: B = regression coefficient; SE = standard error; CI = confidence interval. The model shows a negative and significant effect of Optimism on Jealousy (p < .001).***

Table 3. Regression Analysis Results Regarding the Effect of Optimism and Jealousy Scale Scores on the Emotion Regulation Scale in Athletes

| **Variable** | **B** | **SE** | **t** | **p** | **95% CI Lower** | **95% CI Upper** | **R** | **R²** | **F** | **p (model)** |
| --- | --- | --- | --- | --- | --- | --- | --- | --- | --- | --- |
| **Constant** | –6.8334 | 0.3701 | –18.4631 | <.001 | –7.5604 | –6.1064 | .7853 | .6167 | 437.56 | <.001 |
| Optimism | 2.2014 | 0.0811 | 27.1400 | <.001 | 2.0420 | 2.3607 |  |  |  |  |
| **JSA** | 1.3561 | 0.0625 | 21.6831 | <.001 | 1.2332 | 1.4789 |  |  |  |  |

Note: B = regression coefficient; SE = standard error; CI = confidence interval. Both Optimism and Jealousy are positive and significant predictors of Emotion Regulation (p < .001).

**Table 4.** Hayes PROCESS Model 4 Results Regarding the Total, Direct, and Indirect Effects of Optimism Scale Score on Emotion Regulation in Athletes

| **Effect Type** | **B (Effect)** | **SE / BootSE** | t | p | 95% CI Lower | 95% CI Upper |
| --- | --- | --- | --- | --- | --- | --- |
| Total Effect | 1.4947 | 0.1013 | 14.7521 | < .001 | 1.2957 | 1.6937 |
| Direct Effect | 2.2014 | 0.0811 | 27.1400 | < .001 | 2.0420 | 2.3607 |
| Indirect Effect | –0.7067 | 0.0562 (boot) | — | — | –0.8232 | –0.5982 |

Note: The indirect effect was tested using the Bootstrap method (n = 5000 samples). The 95% confidence interval [-0.8232, -0.5982] did not include zero, indicating a significant negative indirect effect. Because the positive direct effect and negative indirect effect had opposite signs, the optimism model showed an inconsistent/suppressing mediation pattern.

**Table 5. Outcome Model Results for Mediation Model 2: Pessimism** → **Jealousy → Emotion Regulation**

| **Predictor** | Coefficient (B) | **SE** | t-value | p-value | Lower 95% CI | Upper 95% CI |
| --- | --- | --- | --- | --- | --- | --- |
| Constant | -1.3125 | 0.5059 | -2.59 | .0097 | -2.3063 | -0.3187 |
| Pessimism | -2.0644 | 0.4910 | -4.21 | < .001 | -3.0288 | -1.1000 |
| JSA | 3.5138 | 0.3182 | 11.04 | < .001 | 2.8887 | 4.1388 |

Note: In the revised Pessimism model, Pessimism had a negative direct association with AERS scores (B = -2.0644, SE = 0.4910, p < .001), whereas Jealousy positively predicted AERS scores (B = 3.5138, SE = 0.3182, p < .001). The total effect was B = 3.21. Based on the rounded coefficients reported in the retained analysis, the indirect effect through Jealousy was approximately B = 5.27. The original 5,000-sample bootstrap analysis reported that the 95% confidence interval excluded zero; exact bootstrap confidence-limit values were not retained. The opposite signs of the direct and indirect effects indicate an inconsistent mediation pattern.
